# A five arm natural history study of nasal vestibulitis

**DOI:** 10.1002/cam4.5887

**Published:** 2023-04-05

**Authors:** Elizabeth J. Cathcart‐Rake, David Zahrieh, Deanne Smith, Susan Young, Shaylene McCue, Amanda O'Connor, Stephan Thomé, Mario Lacouture, Terra Register, Jill Piens, Bret B. Friday, Charles L. Loprinzi

**Affiliations:** ^1^ Department of Medical Oncology Mayo Clinic Rochester Minnesota USA; ^2^ Department of Health Sciences Research Mayo Clinic Rochester Minnesota USA; ^3^ Department of Medical Oncology Mayo Clinic – Mankato Mankato Minnesota USA; ^4^ Dermatology Service Memorial Sloan Kettering Cancer Institute New York New York USA; ^5^ Department of Medical Oncology Mayo Clinic – Albert Lea Albert Lea Minnesota USA; ^6^ Essentia Health Cancer Center 420 East First St Duluth Minnesota USA

**Keywords:** chemotherapy side effects, nasal vestibulitis, symptom management

## Abstract

**Introduction:**

Nasal symptoms are frequently reported by patients undergoing chemotherapy.

**Methods:**

Eligible patients planning to receive paclitaxel, docetaxel, nab‐paclitaxel, bevacizumab without a concomitant taxane, or “other” (non‐taxane, non‐bevacizumab) chemotherapy regimens were invited to participate in this prospective study. Patients reported nasal symptoms prior to each dose of chemotherapy.

**Results:**

The percentage of patients (95% CI) who reported nasal symptoms was the same for patients who received bevacizumab or nab‐paclitaxel, 82.6% (61.2%, 95.1%). There were no significant differences among the proportions of patients experiencing nasal symptoms within the paclitaxel, nab‐paclitaxel, and bevacizumab cohorts. Patients in the nab‐paclitaxel cohort were more likely to experience symptoms than those in the non‐taxane non‐bevacizumab cohort or docetaxel cohort (*p* = 0.001, *p* = 0.001). Patients in the bevacizumab cohort were more likely to experience nasal symptoms than those in the non‐taxane non‐bevacizumab cohort (*p* = 0.03).

**Conclusion:**

Nasal vestibulitis symptoms are common in patients receiving chemotherapy, especially those receiving paclitaxel, docetaxel, and bevacizumab. Further investigations into treatments of this symptom complex are warranted.

## Graphical abstract

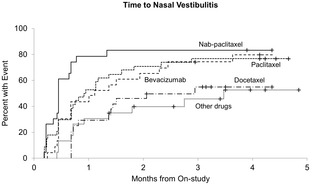

## 1 INTRODUCTION

In 2017, a breast oncology nurse practitioner frequently noted to a senior breast oncology physician that she had observed that patients receiving chemotherapy developed nasal symptoms. The staff physician had not noted this phenomenon; discussions with other breast oncologists did not reveal any recognition of this potential symptom complex in patients with breast cancer. These experiences, shared among colleagues at one Midwest institution, spurred these physician investigators to further evaluate the natural history of these nasal symptoms.

A literature search revealed one manuscript, published in 2015, which described nasal vestibulitis in patients who were receiving cancer therapies, mostly targeted therapies, such as epidermal growth factor inhibitors.^1^ Less than 10% of cases were in patients who were noted to be receiving chemotherapy. Nasal vestibulitis was described as inflammation of the vestibule of the nose, causing nasal pain, bleeding, and discharge.

This led to further evaluation. An initial project involved interviews of patients who had received at least 6 weeks of chemotherapy, asking them whether they had developed any new nasal symptoms.^2^ This work revealed that 41% of patients noted nasal symptoms, which included dryness, pain, bleeding, and scabbing. Over 70% of patients receiving taxanes reported such symptoms; an even higher proportion of patients reported nasal symptoms while on vascular endothelial growth factor (VEGF) therapies. Nasal symptoms were only reported in 41% of symptomatic patients' charts; 61% of symptomatic patients stated that they told their provider about nasal issues. Almost half of the interviewed patients reported that the symptoms were bothersome enough for them that they had used something to treat their symptoms.

A prospective trial was developed to evaluate the incidence of nasal vestibulitis symptoms in five groups of patients: patients receiving paclitaxel, patients receiving docetaxel, patients receiving nab‐paclitaxel, patients receiving bevacizumab (without receiving a taxane), and patients receiving a variety of chemotherapy regimens other than the above‐noted ones. Results from the first three arms of this trial that completed accrual have recently been published, involving approximately 25 patients in each arm (paclitaxel, docetaxel, or non‐taxane non‐bevacizumab chemotherapy).^3^ Seventy‐seven percent of patients receiving paclitaxel reported developing new nasal symptoms, compared with 48% of patients receiving non‐taxane non‐bevacizumab chemotherapy regimens. Patients receiving docetaxel had an incidence closer to the latter group (54%).

The current manuscript describes additional results from the same prospective trial, in patients who received nab‐paclitaxel and bevacizumab and compares these results to the previously‐reported three arms.

## 2 METHODS

The methods of this current clinical trial will be briefly summarized here, as they have been previously reported.^3^

Patients planning to receive at least 2 months of paclitaxel, docetaxel, nab‐paclitaxel, bevacizumab without a concomitant taxane, or “other” (non‐taxane, non‐bevacizumab) chemotherapy regimens were invited to participate in this prospective evaluation of nasal symptoms. At the time of study enrollment, they could not have had a history of more than one nosebleed per month and could not have had nasal dryness, pain, bleeding, or scabbing of 3 or higher on a 0–10 point scale. Patients provided informed consent as mandated by United States federal guidelines. Ethical approval for this study was granted by the Institutional Review Board prior to commencing this study.

Patients completed questionnaires regarding nasal symptoms, at baseline and at the time of each subsequent dose of chemotherapy within the first 4 months following chemotherapy initiation.

Statistical methods utilized in this trial have been previously detailed.^3^ In summary, we evaluated the proportion of patients who reported one or more episodes of new or worsened nasal vestibulitis symptoms, compared with their baseline (termed “treatment‐emergent nasal vestibulitis”) during their chemotherapy course. We estimated the proportion of patients with treatment‐emergent nasal vestibulitis both over the entire group of patients and within each of the patient cohorts. The binomial proportions of patients who reported new or worsening nasal symptoms were estimated with their 95% confidence intervals. *p* values are two sided and reported as continuous values. Additionally, for each cohort of patients, the time to onset of nasal symptoms was estimated using a cumulative incidence function; death and disease progression were treated as competing risks. In order to assess for time to treatment‐emergent nasal vestibulitis, after controlling for several variables (age at baseline [years], smoking history [currently, in the past, never], allergy history [yes, no], and asthma history [yes, no]), a cumulative incidence function regression model was also applied.

## 3 RESULTS

Patients were accrued on the study from March 2018 to February 2021 (baseline data in Table S1).

About 91 out of 127, evaluable patients (71.7%) reported new or worsened nasal symptoms from baseline. The percentage of patients (95% CI) with nasal symptoms was the same for patients who received bevacizumab and nab‐paclitaxel chemotherapy, namely, 82.6% (61.2%, 95.1%). In comparison, 76.5% (58.8%, 89.3%) of patients receiving paclitaxel reported nasal symptoms^3^; among patients who received docetaxel, and non‐taxane non‐bevacizumab chemotherapy, 54.2% (32.8%, 74.5%) and 47.8% (26.8%, 69.4%), respectively, reported new nasal symptoms.^3^

**FIGURE 1:**
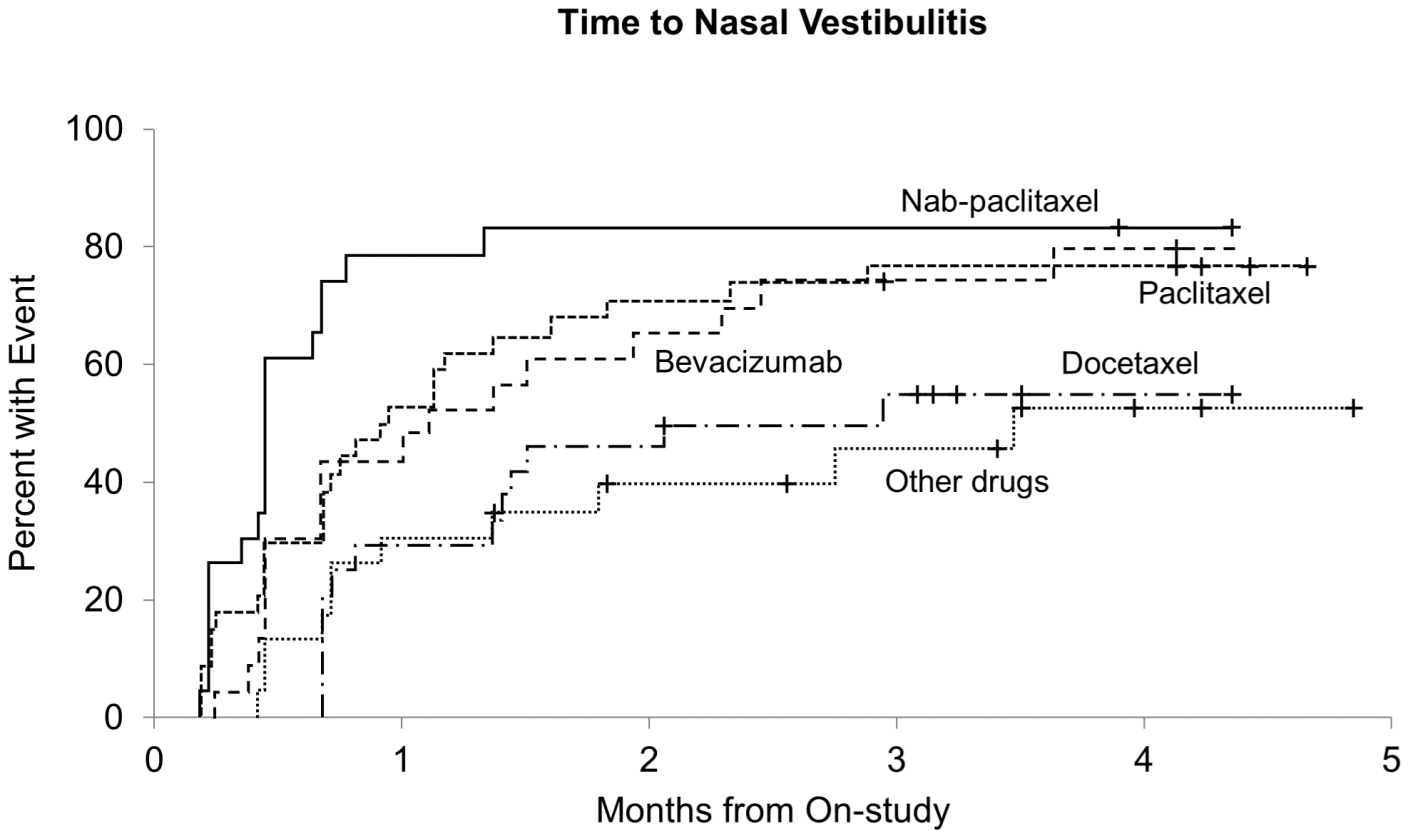
Times to nasal vestibulitis.

Figure 1 illustrates the incidence of nasal vestibulitis over time among patients in each of the five cohorts. There were statistically significant differences in the cumulative incidence of nasal vestibulitis symptoms among the five cohorts of patients (*p* = 0.0002).^3^ Patients receiving paclitaxel had a higher cumulative incidence of new nasal vestibulitis symptoms compared with patients in the non‐taxane non‐bevacizumab cohort (*p* = 0.026),^3^ as well as compared with patients in the docetaxel cohort (*p* = 0.035). Although there was no statistically significant differences among the paclitaxel, nab‐paclitaxel, and bevacizumab cohorts in regards to cumulative incidence, patients receiving nab‐paclitaxel also had a higher cumulative incidence of nasal vestibulitis symptoms when compared with both patients receiving non‐taxane non‐bevacizumab chemotherapy (*p* = 0.001) and patients receiving docetaxel chemotherapy (*p* = 0.001). Patients receiving bevacizumab chemotherapy also had a higher cumulative incidence of nasal vestibulitis compared with patients receiving non‐taxane non‐bevacizumab regimens (*p* = 0.031). While patients receiving bevacizumab had a higher cumulative incidence of nasal symptoms than those receiving docetaxel, this did not reach statistical significance (*p* = 0.067). The significant differences in cumulative incidence remained after controlling for age, smoking history, allergies, and asthma.

**FIGURE 2:**
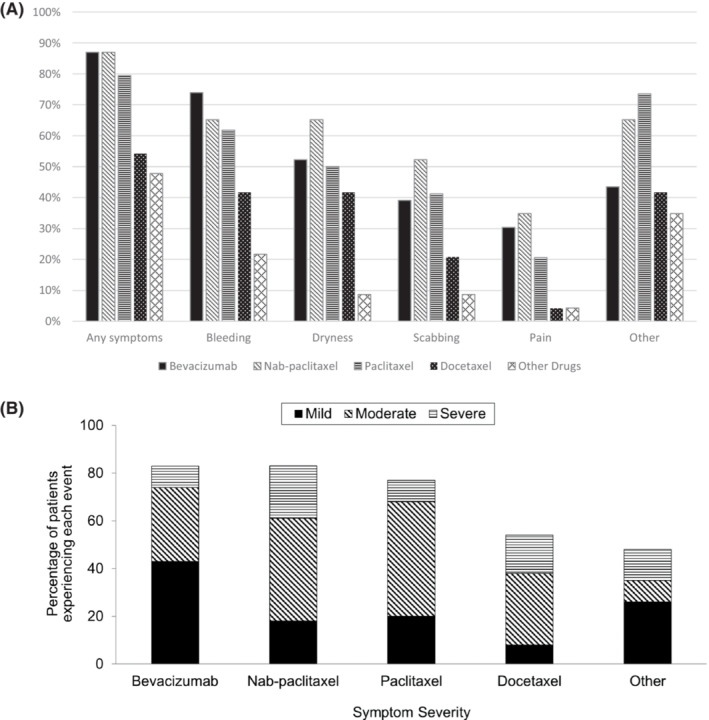
Type of nasal vestibulitis symptoms, as reported by patients at any time during the study (A) and the severity of nasal vestibulitis symptoms, as reported at their worst (B). Note that figure data presented from the paclitaxel, docetaxel, and non‐taxane non‐bevacizumab arms have been previously published; this data is also shown here for purposes of comparison.^3^

Bleeding was the most reported type of nasal symptom, noted by 53.5% of all participants in this study. Data regarding the development of specific types of nasal symptoms, including bleeding, dryness, scabbing, pain, and other, within each study arm are illuminated in Figure 2A. The most commonly reported “other” nasal symptom was nasal drainage (reported in 75.0%, 51 out of the 68 patients, who reported “other” nasal symptoms). The majority of patients reported “moderate” nasal symptoms on a scale from mild to moderate to severe; nasal symptom severity reported by participants within each study arm is illustrated in Figure 2B.

Among the 91 patients who reported nasal symptoms, only 28 patient charts (30.8%) had mention of nasal symptoms, and 10 (11.0%, of patients with nasal symptoms) of these charts reported upon recommended treatments for nasal symptoms, including nasal saline (five patients), a compounded product called rose geranium in sesame oil nasal spray (four patients), and a humidifier (one patient).

## 4 DISCUSSION

This report provides several new insights regarding nasal vestibulitis. First, it prospectively confirms that bevacizumab is associated with substantial nasal vestibulitis, as was suggested in the prior retrospective study.^2^ Second, it supports that substantial nasal vestibulitis occurs in patients receiving standard paclitaxel or nab‐paclitaxel. It is interesting to note that nab‐paclitaxel appears to cause more symptoms than paclitaxel.

Additionally, fewer nasal vestibulitis symptoms were observed in patients undergoing docetaxel chemotherapy, when compared with those undergoing paclitaxel, nab‐paclitaxel, or bevacizumab. In the retrospective study that led to this current report,^2^ data regarding taxanes were combined together. As the majority of the patients in the taxane group received paclitaxel (20 out of the 28 total patients), and only four patients each received docetaxel or nab‐paclitaxel, the retrospective study did not allow comparisons among taxane groups. The present study is the first to prospectively report upon nasal vestibulitis and report it's high incidence among patients receiving paclitaxel and nab‐paclitaxel; this can inform patient‐clinician discussions of chemotherapy toxicities as to the likelihood of nasal symptoms secondary to these agents.

In trying to understand this symptom complex better, it would be interesting to know the incidence of nasal symptoms over 3–4 months in a group of patients who was not receiving chemotherapy. Potentially, such data could be obtained by asking patients to complete questionnaires, as was done in the current trial, while they are receiving hormonal therapy for breast cancer.

Additionally, it would be interesting to know whether nasal vestibulitis symptoms are more prominent at different times of the year, for example during flu season.

With regards to potential treatment of this clinical problem, preliminary data support that a compounded product, rose geranium in sesame oil, appears to be helpful. After some patients reported that this compound improved their nasal symptoms, a retrospective evaluation of its potential benefit among patients undergoing cancer‐directed therapy was conducted. Of 20 patients who used rose geranium nasal spray while undergoing cancer‐directed therapy, all reported symptomatic benefit, and 8 (40%) reported “dramatic” or “complete resolution” of symptoms.^4^ A prospective trial is ongoing (NCT04620369), which randomizes patients undergoing chemotherapy and experiencing nasal vestibulitis to rose geranium in sesame oil nasal spray or nasal saline. This trial should be able to better determine the efficacy of these two products for treating chemotherapy‐induced nasal vestibulitis.

## AUTHOR CONTRIBUTIONS

**Elizabeth J. Cathcart‐Rake:** Conceptualization (equal); data curation (equal); formal analysis (equal); investigation (equal); methodology (equal); project administration (equal); writing – original draft (lead); writing – review and editing (lead). **David Zahrieh:** Formal analysis (lead); writing – review and editing (equal). **Deanne Smith:** Conceptualization (equal); writing – review and editing (equal). **Susan Young:** Data curation (equal); investigation (equal); methodology (equal). **Shaylene McCue:** Formal analysis (equal); writing – review and editing (equal). **Amanda O'Connor:** Formal analysis (equal); writing – review and editing (equal). **Stephan Thomé:** Investigation (equal); writing – review and editing (equal). **Mario Lacouture:** Conceptualization (equal); methodology (supporting); writing – review and editing (equal). **Terra Register:** Investigation (equal). **Jill Piens:** Investigation (equal); writing – review and editing (equal). **Bret B. Friday:** Investigation (equal); writing – review and editing (equal). **Charles L. Loprinzi:** Conceptualization (lead); data curation (equal); formal analysis (equal); funding acquisition (lead); investigation (lead); methodology (lead); project administration (equal); resources (equal); supervision (lead); validation (equal); visualization (equal); writing – original draft (equal); writing – review and editing (equal).

## FUNDING INFORMATION

This work was supported by funding from the Minnesota Cancer Clinical Trials Network (MNCCTN). Funding was provided by the Breast Cancer Research Foundation (BCRF).

## CONFLICT OF INTEREST STATEMENT

The authors do not have affiliations or involvement in any entity with financial interest relevant to the submitted work.

## Supporting information


Table S1

## Data Availability

The data that support the findings of this study are available from the corresponding author upon reasonable request.
