# Supplementary material for: A five arm natural history study of nasal vestibulitis
Source: Cancer Med. 2023 Apr 5;12(8):9650–4. doi: 10.1002/cam4.5887 (PMC10166902; doi:10.1002/cam4.5887)
Supplement: Supplementary file 1 — Table S1 [file CAM4-12-9650-s001.docx]

Table 1. Baseline Patient Characteristics.

|  | *Paclitaxel^1^* | *Docetaxel^1^* | *Nab-paclitaxel* | *Bevacizumab* | *Non-Taxane Non-Bevacizumab Chemotherapy^1^* |
| --- | --- | --- | --- | --- | --- |
| **Number of patients** | 34 | 24 | 23 | 23 | 23 |
| **Age** mean (SD) | 59.4 (9.8) | 61.2 (10.0) | 65.6 (11.7) | 62.0 (13.8) | 60.4 (10.8) |
| **Gender**  Female  Male | 32 (94%)  2 (6%) | 21 (88%)  3 (12%) | 16 (70%)  7 (30%) | 12 (52%)  11 (48%) | 17 (74%)  6 (26%) |
| **Smoking status**  Current  Never | 1 (3%)  22 (67%) | 6 (25%)  15 (65%) | 1 (4%)  9 (41%) | 4 (17%)  11 (58%) | 3 (13%)  9 (41%) |
| **History of seasonal allergies** | 12 (35%) | 9 (38%) | 8 (35%) | 8 (35%) | 4 (17%) |
| **History of asthma** | 6 (18%) | 1 (4%) | 5 (22%) | 2 (8%) | 1 (4%) |
| **Baseline nasal symptoms** | 0 (0%) | 0 (0%) | 2 (8%) | 1 (4%) | 3 (13%) |

1. Data from these three arms previously published: Cathcart-Rake EJ, Zahrieh D, Smith D, et al. Natural history of nasal vestibulitis associated with paclitaxel, docetaxel, and other chemotherapy agents: a Minnesota Cancer Clinical Trials Network (MNCCTN) study. *Support Care Cancer.* 2021;29(11):6253-6258.
